# Atlas and Epitome of Operative Gynecology

**Published:** 1904-05

**Authors:** 


Atlas and Epitome of Operative Gynecology. By Dr. O. Schaeffer, of Heidelberg. Edited, with additions, by J. Clarence Webster, M. D. (Edin.), Professor of Obstetrics and Gynecology in Rush Medical College, University of Chicago. With 42 lithographic plates in colors, many text cuts, a number in colors, and 138 pages of text. Philadelphia, New York and London: W. B. Saunders & Company. 1904. (Cloth, $3.00 net.)

The editor very appropriately says in his preface that students who graduate each year, know less about gynecologic operations than about almost any other department of operative surgery. This is because these clinics are practically valueless to the great majority of students. There is only one branch of operative surgery about which the average student knows less than he does of gynecology, and that is genitourinary work. And yet, they go forth to practise and take charge of gynecologic and genitourinary cases, which sometimes turn out well, not because the practitioner is adept or even superficially versed in either specialty, but because nature is long suffering and is kind.

It is to teach the student something of gynecology that this work is written, and it assuredly fills its mission admirably. The work is not a collection of rewritten articles; it is original, and is based on Dr. Schaeffer's abundant experience, and while it may not be regarded as a comprehensive textbook, it is much more valuable to the student than many works on the subject which are more pretentious in character. The illustrations are of the same excellent general style which marks all these atlases, and no practitioner would be excusable if he seriously blundered after a careful study of the descriptive text and very understandable illustrations. The book is a gem in the chain of volumes which make up the series. N. W. W.

